# Metal(II) Coordination Polymers Derived from Mixed 4-Imidazole Ligands and Carboxylates: Syntheses, Topological Structures, and Properties

**DOI:** 10.3390/polym10060622

**Published:** 2018-06-06

**Authors:** Wei-Dong Li, Jia-Le Li, Xing-Zhe Guo, Zhi-You Zhang, Shui-Sheng Chen

**Affiliations:** 1 School of Chemistry and Chemical Engineering, Fuyang Normal University, Fuyang 236041, China; weidongli2018@163.com (W.-D.L.); lijiale_ljl@163.com (J.-L.L.); zhujjj1993@126.com (X.-Z.G.); fyzhiyouzhang@126.com (Z.-Y.Z.); 2 Coordination Chemistry Institute, State Key Laboratory of Coordination Chemistry, Nanjing University, Nanjing 210093, China

**Keywords:** coordination polymers, crystal structures, photoluminiscent property, adsorption property

## Abstract

Four new metal–organic coordination polymers [Cu(L)(mpa)]·3H_2_O (**1**), [Co(L)(mpa)]·H_2_O (**2**), [Zn(L)(mpa)]·H_2_O (**3**), and [Cd(L)(mpa)(H_2_O)]·H_2_O (**4**) were synthesized by reactions of the corresponding metal(II) salts based on mixed ligands of 1,4-di(1*H*-imidazol-4-yl)benzene (L) and 4-methylphthalic acid (H_2_mpa), respectively. The structures of the complexes were characterized by elemental analysis, FT-IR spectroscopy, and single-crystal X-ray diffraction. Compound **1** exhibits a binodal 4-connected three dimensional (3D) architecture with (6^5^·8)-**CdSO_4_** topology, while complexes **2** and **3** are isostructural and have two-dimensional (2D) layer structure with (4, 4) **sql** topology based on the binuclear metal subunits. Complex **4** has the same 2D layer structure with (4, 4) **sql** topology as complexes **2** and **3**, but the inclined interpenetration of parallel sets of layers result in the formation with 2D + 2D → 3D framework. The activated sample **1** shows selective CO_2_ uptake over N_2_. The photoluminiscent properties together with quantum yield (QY) and luminescence lifetime are also investigated for complexes **3** and **4** in the solid state at room temperature.

## 1. Introduction

**Scheme 1:**
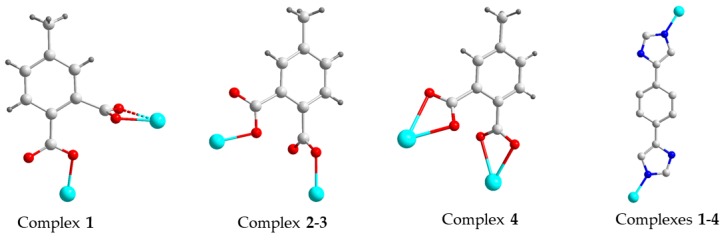
The scheme of coordination modes of H_2_mpa and L ligands in **1–4**.

The design and construction of coordination polymers (CPs), and especially the subclass of metal−organic frameworks (MOFs), can be regarded nowadays as one of the most active research areas in recent years, not only due to their fascinating structural diversities, but also because of their interesting properties and potential applications as functional materials in many fields [1,2,3,4,5,6,7,8,9,10]. In principle, the assembly of MOFs can be influenced by several factors, such as the reaction condition, coordination geometry of the metal ions, nature of anions, flexibility, and coordination modes of the ligands, temperature, solvent, and so on [11,12,13]. Particularly, the key to design of MOFs with such outstanding features mainly depends on the judicious and rational choice of the bridging ligand because it greatly influences the molecular packing arrangement of the compound [14,15,16,17]. The N-donor ligands as well as the polycarboxylates, as two most commonly used organic linkers in the assembly of MOFs, have been widely designed and selected for their various coordination modes, and modifiable backbones. The N-donor ligands with rod-type two-connector between the two terminal coordination groups, for example, 4,4′-bipyridine(bpy), 1,2-bis(4-pyridyl)ethane(bpe), or their analogues, can be employed as ‘pillars’ together with the carboxylate ligand to meet the requirement of coordination geometries of metal ions in the assembly process [18,19,20,21]. More recently, we have designed a novel type of 4-imidazol-containing ligands with versatile coordination modes such as 1,4-di(1*H*-imidazol-4-yl)benzene and 1,3,5-tri(1*H*-imidazol-4-yl)benzene, and successfully constructed a series of porous metal-imidazolate complexes, which exhibit exceptional gas adsorption properties [22,23]. On the other hand, the polycarboxylates are the most extensively studied organic ligands in the construction of MOFs because the carboxylate groups have a high affinity for metal ions and may offer the possibility of incorporating more metal atoms into high-nuclearity clusters [24,25,26]. It is noteworthy that the polycarboxylates and N-donors show different coordination preferences when coordinating with metal ions owing to the different electron configurations of N and O atoms [27,28]. Therefore, the mixed system including multi-N donor and carboxylate ligands can be effectively employed to build diverse coordination polymers due to their favorable compatibility. Following the mixed ligand strategy, we have focused our attention on the reactions of rigid 4-imidazole-containing ligands, together with different carboxylate ligands and different metal salts and synthesized a series of novel MOFs based on the N and O donor mixed spacers [29,30,31,32]. In this contribution, we focus our attention on the study on reactions of ligand 1,4-di(1*H*-imidazol-4-yl)benzene (L) together with 4-methylphthalic acid (H_2_mpa) ligand and metal salts for the assembly of MOFs as an extension of our work. Herein, we report the synthesis and crystal structure of four new coordination polymers [Cu(L)(mpa)]·3H_2_O (**1**), [Co(L)(mpa)]·H_2_O (**2**), [Zn(L)(mpa)]·H_2_O (**3**), and [Cd(L)(mpa)(H_2_O)]·H_2_O (**4**) obtained by reactions of L and H_2_mpa, with corresponding metal salts under hydrothermal conditions, respectively. The scheme of coordination modes of H_2_mpa and L ligands in complexes **1–4** is exhibited in Scheme 1; the various coordination modes of L ligand is also described in detail [32].

## 2. Materials and Methods

### 2.1. Materials and Physical Techniques

The ligand 1,4-di(1*H*-imidazol-4-yl)benzene (L) was prepared as previously described [33]. Other chemical reagents and solvents were purchased commercially and were used as received without further purification. Elemental analyses were performed using a Perkin-Elmer 2400 elemental analyzer (Perkin-Elmer, Inc., Billerica, MA, USA). The infrared spectrum was recorded on a Nicolet Fourier Transform IR (Nicolet Instrument Inc., Madison, WI, USA), and a Nicolet MAGNA-IR 500 spectrometer (Nicolet Instrument Inc., Madison, WI, USA) in the range of 500–4000 cm^−1^ using the KBr disc technique. Thermogravimetric analyses (TGA) were performed on a computer-controlled Perkin-Elmer 7 Series/UNIX TGA7 analyzer (Perkin-Elmer, Inc., Billerica, MA, USA). Gas adsorption experiments were carried out on an Autosorb-iQ gas sorption instrument in Quantachrome Instruments U.S (Quantachrome, Delray Beach, FL, USA). The sample was activated by using the “outgas” function of the surface area analyzer (Quantachrome, Delray Beach, FL, USA) for 24 h at 160 °C. Photoluminescence spectra for the solid samples were recorded with a HORIBA FluoroMax-4 fluorescence spectrophotometer at room temperature. The decay lifetimes were measured with an FLS920P fluorescence spectrometer (Edinburgh Instruments, Edinburgh, UK) in the solid state at room temperature.

### 2.2. Synthesis of [Cu(L)(mpa)]·3H_2_O (**1**)

Reaction mixture of L (21.2 mg, 0.1 mmol), Cu(NO_3_)_2_·3H_2_O (24.2 mg, 0.1 mmol), H_2_mpa (18.0 mg, 0.1 mmol), and H_2_O (8 mL) was adjusted to pH = 7 with 0.5 mol·L^−1^ NaOH solution. The mixture was then sealed into a 16 mL Teflon-lined stainless steel container and heated at 160 °C for 3 days. After cooling to the room temperature, green block crystals of **1** were collected by filtration and washed by water and ethanol for several times with a yield of 72%. Anal. calcd. for C_21_H_22_N_4_O_7_Cu (%): C, 49.85; H, 4.38; N, 11.07. Found: C, 49.68; H, 4.49; N, 10.89. IR (KBr pellet, cm^−1^): 3427 (m), 3140 (m), 1567 (vs), 1544 (vs), 1510 (m), 1396 (vs), 1367 (m), 1221 (w), 1141 (m), 1077 (m), 952 (w), 844 (s), 808 (m), 796 (m), 678 (w), 647 (w), 625 (w), 535 (w), 438 (w).

### 2.3. Synthesis of [Co(L)(mpa)]·H_2_O (**2**)

Complex **2** was obtained by the same hydrothermal procedure as that used for the preparation of **1** using Co(NO_3_)_2_·6H_2_O (29.1 mg, 0.1 mmol) instead of Cu(NO_3_)_2_·3H_2_O. After the reaction mixture was cooled down to room temperature, purple crystals of **2** were collected with a yield of 65%. Anal. calcd. for C_21_H_18_N_4_O_5_Co(%): C, 54.20; H, 3.90; N, 12.04. Found: C, 54.01; H, 4.12; N, 11.92. IR (KBr pellet, cm^−1^): 3488 (m), 3394 (m), 3145 (m), 2851 (m), 1645 (m), 1577(s), 1540 (vs.), 1496 (s), 1415 (vs.), 1362 (s), 1268 (m), 1176 (w), 1166 (w), 1139(s), 1129(s), 1079 (m), 970 (m), 840 (s), 823 (s), 801 (m), 685 (w), 650 (s), 637 (m), 529 (w), 461 (w).

### 2.4. Synthesis of [Zn(L)(mpa)]·H_2_O (**3**)

Complex **3** was obtained by the same hydrothermal procedure as that used for the preparation of **1** using Zn(NO_3_)_2_·6H_2_O (29.7 mg, 0.1 mmol) instead of Cu(NO_3_)_2_·3H_2_O. After the reaction mixture was cooled down to room temperature, colorless crystals of **3** were collected with a yield of 72%. Anal. calcd. for C_21_H_18_N_4_O_5_Zn(%): C, 53.46; H, 3.85; N, 11.88. Found: C, 53.31; H, 3.96; N, 11.42. IR (KBr pellet, cm^−1^): 3485 (m), 3390 (w), 3134 (m), 2852 (m), 1647 (w), 1587 (vs.), 1546 (vs.), 1479 (s), 1415 (vs.), 1356 (s), 1270 (w), 1140 (s), 1081 (m), 971 (m), 949 (m), 840 (s), 799 (s), 685 (w), 649 (s), 529 (m), 476 (w), 485 (m).

### 2.5. Synthesis of [Cd(L)(mpa)(H_2_O)]·H_2_O (**4**)

A mixture of CdCl_2_·2.5H_2_O (22.8 mg, 0.1 mmol), L (21.0 mg, 0.1 mmol), 4-H_2_mpa (18.0 mg, 0.1 mmol) and NaOH (12.0 mg, 0.3 mmol) in 10 mL H_2_O was sealed in a 16 ml Teflon lined stainless steel container and heated at 180 °C for 3 d. Brown block crystals of **4** were collected in 68% yield after being washed with water and ethanol several times. Anal. calcd for C_21_H_20_N_4_O_6_Cd (%): C, 46.99; H, 3.76; N, 10.44. Found: C, 46.81; H, 3.90; N, 11.42. IR (KBr pellet, cm^−1^): 3135 (m), 2362 (w), 1593 (m), 1552 (vs), 1505 (m), 1395 (vs), 1270 (w), 1221 (w), 1172 (w), 1138(s), 1074 (m), 945 (s), 842 (s), 800 (m), 719 (w), 680 (w), 649 (m), 618 (m), 563 (w), 416 (w).

### 2.6. Crystallographic Data Collection and Refinements

The data collections for **1–4** were carried out on a Bruker Smart Apex CCD (Charge Coupled Device) area-detector diffractometer using graphite-monochromated Mo K*α* radiation (*λ* = 0.71073 Å) at 23(2) °C. The diffraction data was integrated by using the SAINT program [34]. Semi-empirical absorption corrections were applied using the SADABS program [35]. The structures were solved by direct methods and all nonhydrogen atoms were refined anisotropically on *F*^2^ by the full-matrix least-squares technique using the SHELXL-97 crystallographic software package [36]. The hydrogen atoms were generated geometrically. The details of the crystal parameters, data collection, and refinements for the complexes are summarized in Table 1, selected bond lengths and angles with their estimated standard deviations are listed in Table S1. The hydrogen atoms of solvent water molecules in **1** and **2** were found directly from the differential Fourier map. All the other hydrogen atoms were generated geometrically except that the hydrogen atoms that were part of water molecules were not identified. Crystal data and details of the data collection and structure refinements for **1–4** are summarized in Table 1. CCDC (Cambridge Crystallographic Data Centre)-1836731, 1836732, 1836733 and 1836734 for **1**, **2**, **3**, and **4**, respectively. These data can be obtained free of charge at www.ccdc.cam.ac.uk/conts/retrieving.html or from the Cambridge Crystallographic Data Centre, 12, Union Road, Cambridge CB2 1EZ, UK; fax: (internet) +44-1223/336-033; email: deposit@ccdc.cam.ac.uk.

## 3. Results

### 3.1. Structural Descriptions

#### 3.1.1. Structure of [Cu(L)(mpa)]·3H_2_O (**1**)

**Figure 1:**
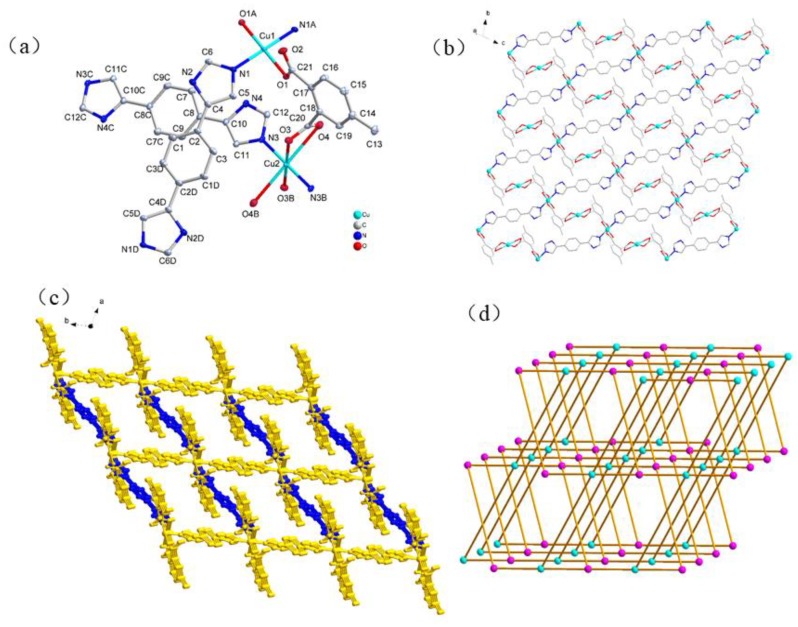
(**a**) The coordination environment of the Cu(II) atoms in **1** with the ellipsoids drawn at the 30% probability level. The hydrogen atoms and free water molecules are omitted for clarity. Symmetry code: A -x, 2 - y, 1 - z, B -x, 2 - y, -z; (**b**) 2D network of **1**; (**c**) 3D structure of **1** constructed from the 2D networks (yellow) pillared by L ligands (blue); (**d**) schematic representation of the binodal (4, 4)-connected 3D framework of **1** with **CdSO_4_** topology with Schläfli symbol (6^5^·8).

The result of X-ray diffraction analysis revealed that complex **1** crystallizes in a triclinic form with space group of P-1 (Table 1), and the asymmetric unit consists of two kinds of Cu(II) atoms, namely, both of Cu(II) atoms sitting on a special position with a half of occupancy, two halves of the L ligand, one completely deprotonated mpa^2^^−^ ligand, and three free water molecules as shown in Figure 1a. The Cu1 atom is four-coordinated in square-planar coordination geometry, which is surrounded by two carboxylate oxygen atoms (O1, O1A) from two individual mpa^2^^−^ ligands, and two nitrogen atoms (N1, N1A) from two different L ligands. The Cu2 atom exhibits a distorted octahedral environment in which the basal plane consists of the four O atoms (O3, O4, O3B, and O4B) from carboxylate groups of mpa^2^^−^ ligands while the axial position is filled by two symmetrical 4-imidazolyl N atoms (N3 and N3B) from two different L ligands. When the connections via the L ligands (containing N1 and N2 atoms) are ignored, the Cu(II) atoms are linked through the other L ligands (containing N3 and N4 atoms) together with mpa^2^^−^ ligands, leading to the formation of a two-dimensional (2D) coordination network (Figure 1b). The just ignored L ligands acts as two-connector pillars to link Cu(II) atoms of adjacent 2D nets to form three-dimensional (3D) pillar-layered frameworks with void channels along the *c* axis (Figure 1c). The total void value of the channel without water guests is estimated (by PLATON) [37] to be 187.1 Å, approximately 17.2% of the total crystal volume of 1090.1 Å. Topological analysis was carried out to analyze the structure of **1**. As discussed above, each mpa^2^^−^ and L ligand link two Cu(II) atoms, respectively, and accordingly, the mpa^2^^−^ and L can be regarded as 2-connected nodes, respectively. As for Cu1 and Cu2 atoms, both of them in turn link two mpa^2^^−^ and two L ligands; hence, they can be treated as 4-connectors. According to the simplification principle, the resulting structure of the complex **1** is a bimodal (4, 4)-connected net with a Schläfli symbol (6^5^·8), which has been referred to by O’Keeffe and Wells as the CdSO_4_ notation (Figure 1d) [38].

#### 3.1.2. Structures of [Co(L)(mpa)]·H_2_O (**2**) and [Zn(L)(mpa)]·H_2_O (**3**)

**Figure 2:**
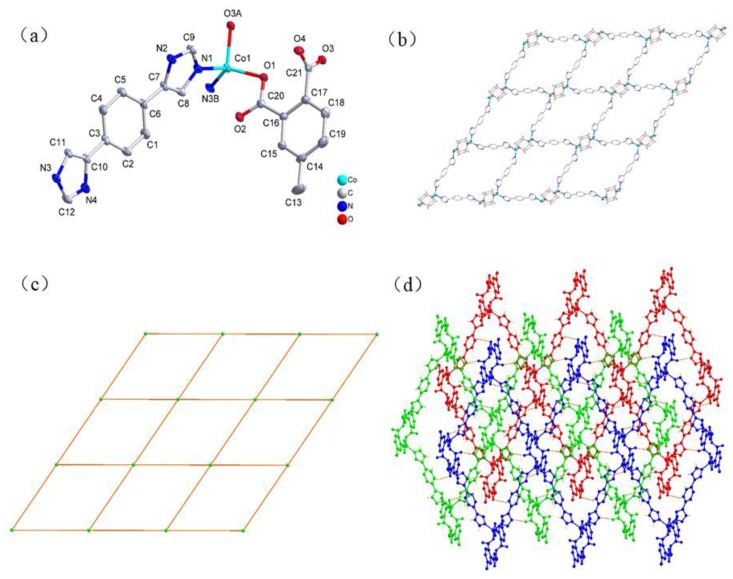
(**a**) The coordination environment of the Co(II) atoms in **2** with the ellipsoids drawn at the 30% probability level. The hydrogen atoms and free water molecules are omitted for clarity. Symmetry code: A -x, 2 - y, 2 - z, B -0.5 - x, 0.5 + y, 0.5 - z; (**b**) 2D network of **2**; (**c**) schematic representation of 2D network with (4, 4) topology in complex **2**; (**d**) 3D framework of **2** linked by hydrogen bonds indicated by a dashed line (different color represents 2D layer).

As Co(NO_3_)_2_·6H_2_O and Zn(NO_3_)_2_·6H_2_O, instead of Cu(NO_3_)_2_·3H_2_O, was used in the reaction of **1,** respectively, **2** and **3** with different structures were isolated. Complexes **2** and **3** crystallize in the same monoclinic P2_1_/n space group with similar cell parameters (Table 1), and the results of X-ray crystallographic analysis indicate that they are isomorphous and isostructural. Thus, as a typical example, only the structure of **2** is described here in detail. The asymmetric unit of **2** contains one unique Co(II) atom coordinated by two nitrogen atoms (N1 and N3B) from two different L ligands and two carboxylate oxygen atoms (O1 and O3A) from two different mpa^2^^−^ ligands, thereby forming a four-coordinated tetrahedral coordination geometry with a N_2_O_2_ donor set (Figure 2a). The Co–O bond lengths are 1.974(19) and 2.002(18) Å, and Co–N ones are 2.036(2) and 2.015(2) Å (Table S1), which are consistent with the reported four-coordinated Co(II) complexes with O and N donors [39]. The mpa^2^^−^ ligand connects two Co(II) atoms using its two adjacent carboxylate groups with *µ*_1_-*η*^1^*:η*^0^-monodentate coordination mode. Interestingly, four carboxylate groups from two different mpa^2^^−^ ligands together with two Co(II) atoms form a Co_2_(COO)_2_ binuclear SBU with a Co···Co distance of 5.45 Å. Each such SBU links four identical ones through four L ligands via Co–N coordination leading to formation of a 2D network with (4, 4) **sql** topology by taking the binuclear SBUs as 4-connecting nodes and the L ligands as linkers (Figure 2b,c). The 2D layers are further linked together via extensive intermolecular N–H···O, O–H···O and C–H···O hydrogen bonds to generate a 3D structure (Figure 2d).

#### 3.1.3. Structure of [Cd(L)(mpa)(H_2_O)]·H_2_O (**4**)

**Figure 3:**
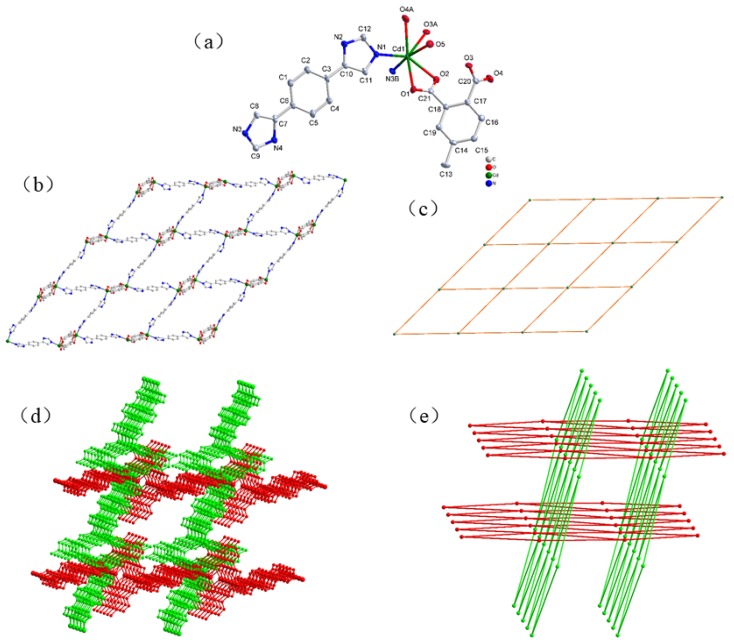
(**a**) The coordination environment of the Cd(II) atoms in **4** with the ellipsoids drawn at the 30% probability level. The hydrogen atoms and water molecules are omitted for clarity. Symmetry code: A 1 − x, −0.5 + y, 0.5 − z, B −x, −y, 1 − z; (**b**) 2D network of **4**; (**c**) schematic representation of 2D network with (4, 4) topology in complex **4**; (**d**) mutual interpenetration of parallel sets of layers in **4** (different color represents 2D layer); (**e**) schematic illustration of 2D + 2D → 3D mutual inclined interpenetration in **4** (different color represents schematic illustration of 2D).

To further investigate the effect of metal salt, the Cu(NO_3_)_2_·3H_2_O was changed to CdCl_2_·2.5H_2_O, and complex **4** with a different structure was obtained. The asymmetric unit of **1** contains one Cd(II) ion, one L ligand, one mpa^2^^−^ ligand, one coordinated, and one lattice water molecules. The Cd1 atom is seven-coordinated by two N atoms (N1, N3B) from two different L ligands, four O atoms (O1, O2, O3A, and O4A) from two pairs of chelating carboxylate groups of two different mpa^2^^−^ ligands and one O atom (O5) from coordinated water molecule (Figure 3a). The average Cd1−O and Cd1−N distances are 2.462(3) and 2.293(3) Å, respectively, and the coordination angles around Cd1 varies from 53.39(9) to 169.70(10)° (Table S1). Similar to the complexes **2** and **3**, both of adjacent carboxylate groups from the mpa^2^^−^ ligand connect two Cd(II) atoms in *µ*_1_-*η*^1^*:η*^1^-chelating coordination mode, and four carboxylate groups from two different mpa^2^^−^ ligand connect two Cd(II) atoms to form a Cd_2_(COO)_2_ binuclear SBU with Cd···Cd distance of 5.24 Å. The binuclear SBUs is employed as a 4-connecting node to connect other four identical ones by four linear L ligands, forming the 2D network with (4, 4) topology by taking the binuclear SBUs as 4-connecting nodes and the L ligands as linkers (Figure 3b,c). In **4**, two L ligands and two Cd(II) ions form a macrocycle through the coordination bonds, where the lateral distances of Cd_2_(COO)_2_ binuclear SBU is 18.74 Å, and the through-space apertures within a single layer submotif measure 13.88 × 34.82 Å, with the angles among SBU nodes of 43.45° and 136.55°. Thus, the shape of this macrocycle is a rhombus. The large rectangular windows within the layers permit mutual inclined interpenetration of parallel sets of layers, forming the 2D + 2D → 3D framework (Figure 3d,e) [40,41].

### 3.2. Thermal Analyses and X-ray Powder Diffraction Analyses

Thermogravimetric analysis (TGA) was employed on complexes **1–4** in order to ascertain the stability, and the results are shown in Supporting Information Figure S1. A total weight loss of 10.25% was observed for **1** in the temperature range of 75–140 °C, which is attributed to the loss of three lattice water molecules (calc. 10.67%), and the decomposition of the residue occurred at 235 °C. For **2**, the first weight loss of 3.62% in 200–275 °C indicated the exclusion of one free lattice water molecule (calc. 3.87%), and the residue is stable up to about 330 °C. A total weight loss of 3.95% was observed for **3** in the temperature range of 180–230 °C, which is attributed to the loss of three coordinated and one free water molecules (calc. 3.82%), and the residue is stable up to about 260 °C. A total weight loss of 6.55% was observed for **4** in the temperature range of 50–105 °C, which is attributed to the loss of water molecules (calc. 6.71%), and the residue is stable up to about 305 °C. The pure phase of coordination polymers was proved by the powder X-ray diffraction (PXRD), where the patterns of, as-synthesized, **1–4** are consistent with the corresponding simulated ones (Figure S3), indicating the phase purity of the sample. The further stability information about the porous structure is required in order to make gas adsorption measurements for the porous materials of **1**. After heating of **1** at 160 °C for 24 h in vacuum, the guest water molecules were removed. The PXRD patterns at varied temperature show that the framework of **1** is retained (Figure S2), indicating that complex **1** has permanent porosity after evacuation.

### 3.3. Diffuse Reflectance Spectra

**Figure 4:**
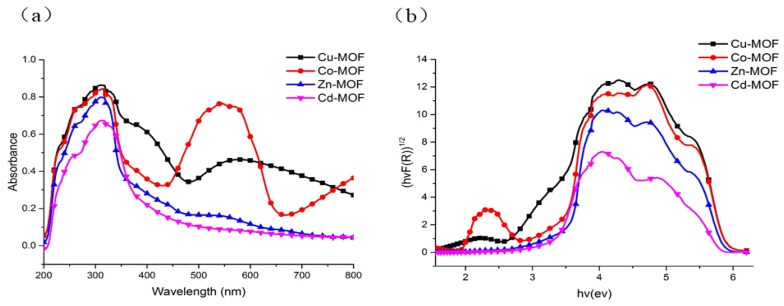
(**a**) The solid-state diffuse reflectance UV-Vis spectra for the complexes **1–4**; (**b**) The result of the right picture was treated with the Kubelka–Munk function.

The solid state diffuse reflectance UV-Vis (Ultraviolet-visible) spectra at room temperature were recorded for the as-synthesized samples of **1–4** (Figure 4a). To study the conductivity of the complexes, the solid state diffuse reflectance UV-Vis spectrum of complexes were applied to calculate the band gap E_g_, which was determined as the intersection point between the energy axis (hν) and the line derived from the linear portion of the absorption edge in a plot of the K–M (Kubelka–Munk) theory F vs. the incident photon energy hν [42]. UV-visible spectra of coordination polymers **1**−**2** have absorptions in the UV region as well as in the visible region, absorptions in the UV region are due to ligand, and in the visible region are due to d−d transitions (^2^E_g_ to ^2^T_2g_) of copper(II) compounds [43,44]. Coordination polymers **3** and **4** shows an absorption in UV-region due to a ligand, and it does not absorb in the visible region because of the A_1g_ ground state. As shown in Figure 4b, the optical band gaps obtained by extrapolation of the linear portion of the diffuse reflectance spectra are estimated as 3.32, 3.55, 3.91, and 3.90 eV for complexes **1–4**, respectively, indicating the existence of optical direct band gap and the characteristic of semiconductivity, which have potential application in the field of semiconductor [45,46,47].

### 3.4. Photoluminescent Property

**Figure 5:**
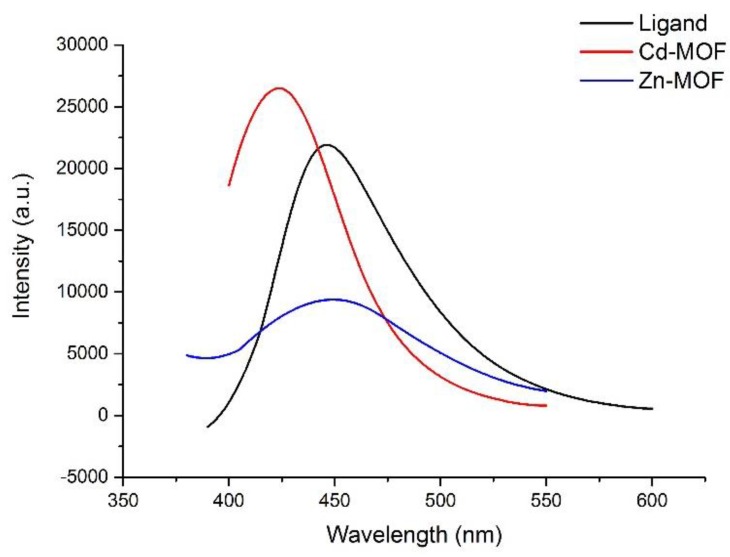
Emission spectra of complexes **3** and **4** together with L ligand.

**Figure 6:**
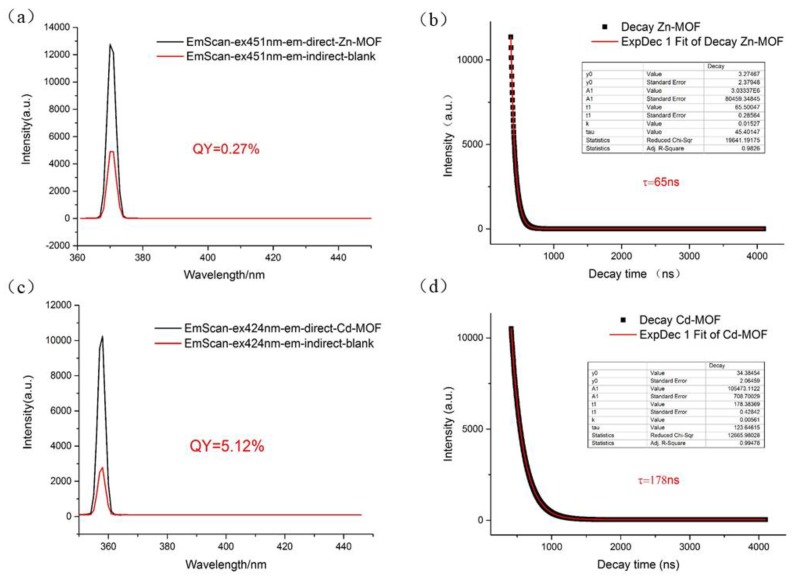
The QY and decay curves of compounds **3** (**a** and **b**) and **4** (**c** and **d**).

Inorganic−organic hybrid coordination polymers have been reported to have the ability to adjust the emission wavelength of organic materials through incorporation of metal centers, especially for d^10^ metal centers [48,49]. The fluorescence properties of the crystalline materials **3** and **4**, as well as the free L ligand have been investigated in the solid state, as depicted in Figure 5. The free L ligand shows emission band at 450 nm upon excitation at 370 nm, which may be attributed to π* → π transition of the intraligands [50,51]. As previously reported [52,53], the fluorescence emission of solid-state benzenecarboxylate ligands can be assigned to the π* → n transition. Comparatively, the fluorescent emission of benzene–dicarboxylate ligands resulting from the π* → n transition is weak in comparison to that of the π* → π transition of the conjugated aromatic ligands such as the L ligand, so benzene–carboxylate ligands almost have little contribution to the fluorescent emission of d^10^ complexes [54,55]. Figure 6 shows that compounds **3** and **4** exhibit blue photoluminescence with emission maxima at 446 and 424 nm upon excitation at 370 and 357 nm, respectively. In contrast to the free L ligand, the emission bands of complexes **3** and **4** are 4nm red-shifted and 22 nm blue-shifted respectively. The emissions of these complexes may be assigned to the cooperative effects of intraligand transition and differences in the coordination environments [56,57]. In addition, it is noteworthy that the enhancement of luminescence for the complex **4** compared with the free ligand and **3** under the same conditions may mainly originate from the coordination interactions between the metal Cd(II) atom and the ligand, which enhanced its conformational rigidity and then decreased the nonradiative energy loss [58].

In order to further study the luminescence properties, study of the corresponding quantum yield (QY) and decay lifetimes were carried out for compounds **3** and **4** together with L ligand. The QY values of compounds **4** (QY_4_ = 5.12%) was higher than complex **3** (QY**_3_** = 0.27%). The high QY values of compounds **4** is probably attributed to the immobilization of the L ligand as it is strongly coordinated to metal Cd(II) ions that effectively increase the rigidity of the ligands and to the low-dimensional structure with a π-conjugated system that decreases the molecular band gap [59,60]. In addition, the luminescence decay curves can be fitted by an exponential function as *I*(t) = A exp(−*t*/*τ*). The longest luminescence lifetime of compound **4** reaches 178 ns, higher than complex **3**, but much shorter than the ones resulting from a triplet state (>10^−3^ s), so the emissions should arise from a singlet state [61,62]. Therefore, the good photoluminescence property of **4** indicates that it could be potentially used as luminescent material.

### 3.5. Gas Sorption Property

**Figure 7:**
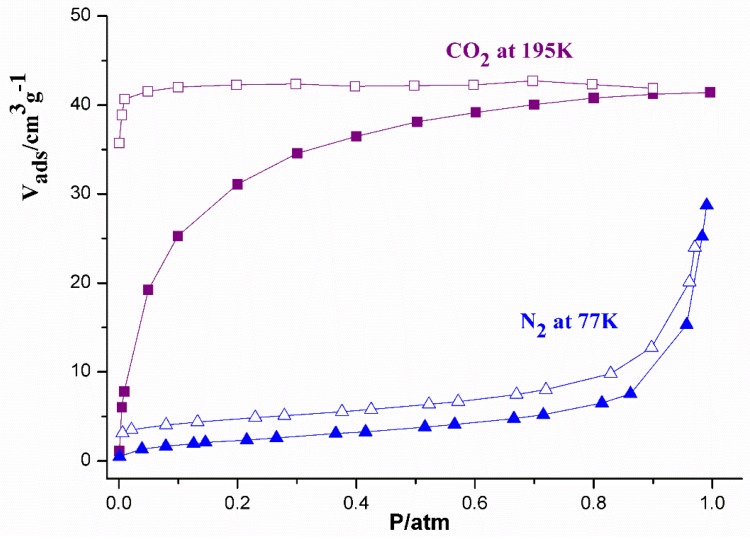
Gas adsorption isotherms of **1′**: filled shape, adsorption; open shape, desorption.

Crystal structure analysis shows that the 3D framework structure in **1** has 17.2% void volume resided by lattice water molecules. The framework of **1** can maintain its porous framework after evacuation, as can be confirmed by TGA and PXRD measurements (Figures S1 and S2). The desolvated solid with 17.2% free void volume in **1** can be employed as microporous material to adsorb gases. Therefore, a further study was subsequently undertaken to investigate the gas adsorption property of the desolvated sample **1′**. The N_2_ and CO_2_ sorption isotherms in Figure 7 shows the microporous material of **1′** exhibit CO_2_ adsorption property, but no N_2_ uptake was observed at 77 K for **1′** in the low-pressure region, and only shows 28.73 cm^3^/g at 1 atm, which exhibits type III sorption profiles, suggesting that only surface adsorption occurs [63]. The CO_2_ gas adsorption isotherms of **1′** display a steep rise at the relative low pressure region which can be categorized as reversible type-I, exhibiting a typical permanent microporosity, while the CO_2_ gas desorption isotherm of **1′** shows pronounced hysteresis as illustrated in Figure 7. The adsorption amount, 9.7 wt% of CO_2_ at 195 K and 1 atm (49.71 cm^3^/g at standard temperature and pressure (STP)), corresponds to 1.1 CO_2_ molecule per formula unit of the solid **1′**. The distinct difference of adsorption capacity between CO_2_ and N_2_ for **1′** must be mainly associated with the smaller kinetic diameters of CO_2_ (3.3 Å) than that of N_2_ (3.64 Å), and the Quadrupole Moment of CO_2_ (−1.4 × 10^−39^ cm^2^) is the important factor that affects the distinct difference in adsorption capacity [64,65].

## 4. Conclusions

The coordination polymers based on mixed multi-N-donor and polycarboxylate auxiliary ligands were synthesized by reactions of the different metal salts under hydrothermal methods. The nature of different metal centers are key factors to construct diverse frameworks. The complex **1** is a rare binodal (4, 4)-connected 3D **CdSO_4_** architecture. Complexes **2** and **3** are isomorphous and isostructural, possessing 2D networks with (4, 4) **sql** topology based on the [M(COO)_2_] SBUs, while complex **4** has 2D **sql** layer motifs which aggregate into a 2D + 2D → 3D mutually inclined interpenetrated framework. The UV-vis absorption spectra of **1–4** are discussed. Moreover, complexes **3** and **4** exhibit intense fluorescence emission and **1** shows CO_2_ gas sorption property. The results imply that not only the N-donor imidazolyl ligands as well as the carboxylate ligand possess variable coordination modes in construction of MOFs, but also show the nature of metal ions and reaction conditions play crucial roles in modulating the formation of the resulting coordination complexes; the combination of rigid N-donor ligand with multi-carboxylate is a good choice for the construction of MOFs with specific structures and properties.

## Figures and Tables

**Table 1:** Crystallographic data and structure refinement details for complexes **1**–**4**.

	1	2	3	4
Empirical formula	C_21_H_22_N_4_O_7_Cu	C_21_H_18_N_4_O_5_Co	C_21_H_18_N_4_O_5_Zn	C_21_H_20_N_4_O_6_Cd
Formula weight	505.97	465.32	471.75	536.78
Temperature/K	296(2)	296(2)	296(2)	296(2)
Crystal system	Triclinic	Monoclinic	Monoclinic	Orthorhombic
Space group	*P*-1	*P*2_1_/n	*P*2_1_/n	*P*bca
*a*/Å	10.1312(4)	11.6926(10)	11.8456(13)	10.8135(5)
*b*/Å	10.7982(4)	16.4817(14)	16.6553(18)	13.8757(7)
*c*/Å	11.7947(5)	11.8661(10)	11.8476(11)	27.2928(12)
*α*/°	105.8050(10)	90	90	90
*β*/°	113.9580(10)	112.9970(10)	114.445(3)	90
*γ*/°	97.6470(10)	90	90	90
*V* (Å^3^)	1090.14(7)	2105.0(3)	2127.9(4)	4095.1(3)
Z, Dcalc/(Mg/m^3^)	2, 1.541	4, 1.468	4, 1.466	8, 1.728
*F*(000)	522	956	960	2128
*θ* range/°	2.97–27.59	2.07–27.46	2.25–26.02	2.40–26.02
Reflections collected	22902	12581	25416	46938
Independent reflections	5040	4738	4166	4030
Goodness-of-fit on *F*^2^	1.053	1.019	1.059	1.096
*R*_1_ [*I* > 2σ (*I*)] ^a^	505.97	465.32	471.75	536.78
*wR*_2_ [*I* > 2σ (*I*)] ^b^	296(2)	296(2)	296(2)	296(2)

^a^*R*_1_ = Σ||*F*_o_| − |*F*_c_||/Σ|*F*_o_|; ^b^ *wR*_2_ = |Σ*w*(|*F*_o_|^2^ − |*F*_c_|^2^)|/Σ|*w*(*F*_o_)^2^|^1/2^, where *w* = 1/[*σ*^2^(*F*_o_^2^) + (*aP*)^2^ + *bP*]. P = (*F*_o_^2^ + 2*F*_c_^2^)/3.

## Supplementary Materials

The following are available online at http://www.mdpi.com/2073-4360/10/6/622/s1, Figure S1: TGA, Figure S2: PXRD, Table S1: Selected bond lengths and bond angles.
