# Supplementary material for: Metal(II) Coordination Polymers Derived from Mixed 4-Imidazole Ligands and Carboxylates: Syntheses, Topological Structures, and Properties
Source: Polymers (Basel). 2018 Jun 6;10(6):622. doi: 10.3390/polym10060622 (PMC6403557; doi:10.3390/polym10060622)
Supplement: Supplementary file 1 [file polymers-10-00622-s001.pdf]

Metal(II) Coordination Polymers Derived from Mixed 4-Imidazole Ligands and Carboxylates: Syntheses, Topological Structures and Properties

Table S1 Selected bond lengths [Å] and bond angles [º] for complexes **1 - 4**

**1**			
Cu(1)-N(1)	1.964(2)	Cu(1)-O(1)	1.9663(17)
Cu(2)-N(3)	1.955(2)	Cu(2)-O(3)	2.0448(19)
N(1)#1-Cu(1)-N(1)	180.000(1)	N(1)#1-Cu(1)-O(1)	90.10(8)
N(1)-Cu(1)-O(1)	89.90(8)	O(1)-Cu(1)-O(1)#1	180.00(10)
N(3)#2-Cu(2)-N(3)	180.000(1)	N(3)#2-Cu(2)-O(3)#2	90.97(8)
N(3)-Cu(2)-O(3)#2	89.03(8)	N(3)#2-Cu(2)-O(3)	89.03(8)
N(3)-Cu(2)-O(3)	90.97(8)	O(3)#2-Cu(2)-O(3)	180.00(11)
**2**			
Co(1)-O(1)	1.9740(19)	Co(1)-O(3)#3	2.0023(18)
Co(1)-N(3)#4	2.015(2)	Co(1)-N(1)	2.036(2)
O(1)-Co(1)-O(3)#3	101.16(8)	O(1)-Co(1)-N(3)#4	122.83(9)
O(3)#3-Co(1)-N(3)#4	109.42(8)	O(1)-Co(1)-N(1)	114.96(8)
O(3)#3-Co(1)-N(1)	102.45(8)	N(3)#4-Co(1)-N(1)	104.17(9)
**3**			
Zn(1)-O(1)	1.948(3)	Zn(1)-O(3)#5	1.992(3)
Zn(1)-N(3)#6	2.009(3)	Zn(1)-N(1)	2.026(3)
O(1)-Zn(1)-O(3)#5	104.29(11)	O(1)-Zn(1)-N(3)#6	120.17(13)
O(3)#5-Zn(1)-N(3)#6	110.62(12)	O(1)-Zn(1)-N(1)	111.76(12)
O(3)#5-Zn(1)-N(1)	103.92(12)	N(3)#6-Zn(1)-N(1)	105.03(13)
**4**			
Cd(1)-N(1)	2.287(3)	Cd(1)-N(3)#7	2.298(3)
Cd(1)-O(2)	2.349(3)	Cd(1)-O(4)#8	2.399(3)
Cd(1)-O(3)#8	2.509(3)	Cd(1)-O(5)	2.517(3)
Cd(1)-O(1)	2.537(3)		
N(1)-Cd(1)-N(3)#7	92.13(11)	N(1)-Cd(1)-O(2)	138.39(10)
N(3)#7-Cd(1)-O(2)	92.99(10)	N(1)-Cd(1)-O(4)#8	83.57(10)

N(3)#7-Cd(1)-O(4)#8	112.10(11)	O(2)-Cd(1)-O(4)#8	131.41(10)
N(1)-Cd(1)-O(3)#8	132.28(10)	N(3)#7-Cd(1)-O(3)#8	87.17(10)
O(2)-Cd(1)-O(3)#8	89.22(9)	O(4)#8-Cd(1)-O(3)#8	53.36(9)
N(1)-Cd(1)-O(5)	97.21(11)	N(3)#7-Cd(1)-O(5)	169.70(10)
O(2)-Cd(1)-O(5)	77.14(10)	O(4)#8-Cd(1)-O(5)	73.45(10)
O(3)#8-Cd(1)-O(5)	89.73(9)	N(1)-Cd(1)-O(1)	85.07(10)
N(3)#7-Cd(1)-O(1)	100.31(10)	O(2)-Cd(1)-O(1)	53.39(9)
O(4)#8-Cd(1)-O(1)	145.93(10)	O(3)#8-Cd(1)-O(1)	141.92(9)
O(5)-Cd(1)-O(1)	76.28(10)		

symmetry codes: #1 -x,-y+2,-z+1, #2 -x,-y+2,-z, #3 -x,-y+2,-z+2, #4 -x-1/2,y+1/2,-z+1/2, #5 -x+2,-y+2,-z+1, #6 -x+5/2,y+1/2,-z+5/2, #7 -x+1,y-1/2,-z+1/2, #8 -x,-y,-z+1.

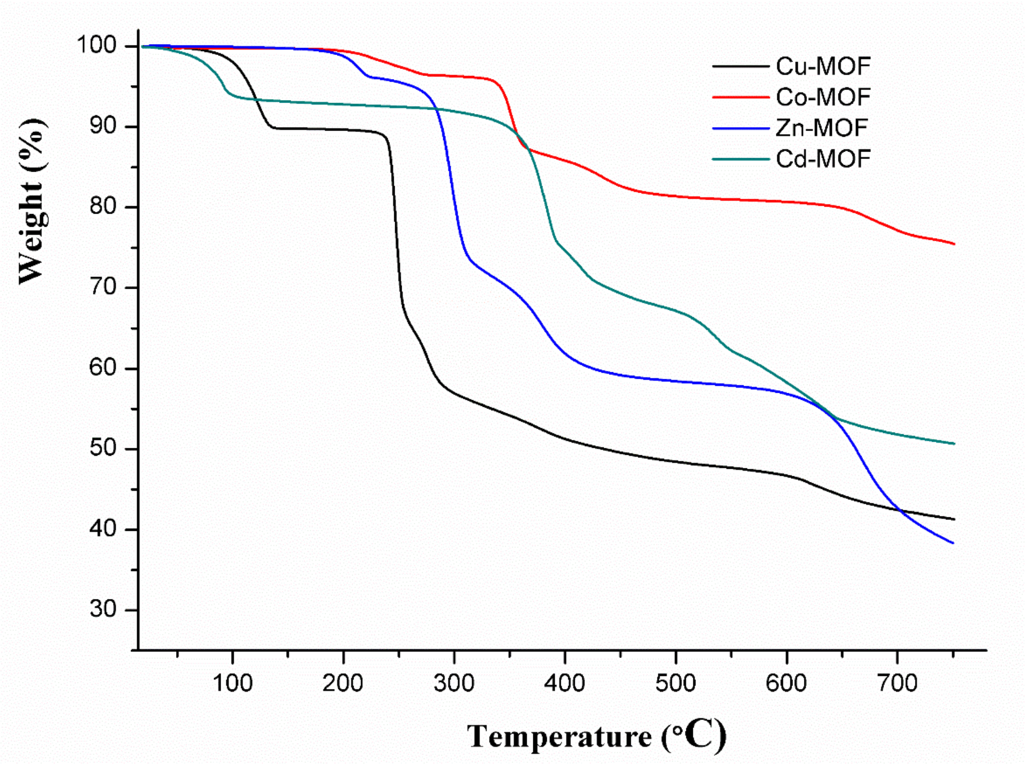

Figure S1. The TG curves of complexes **1 - 4**.

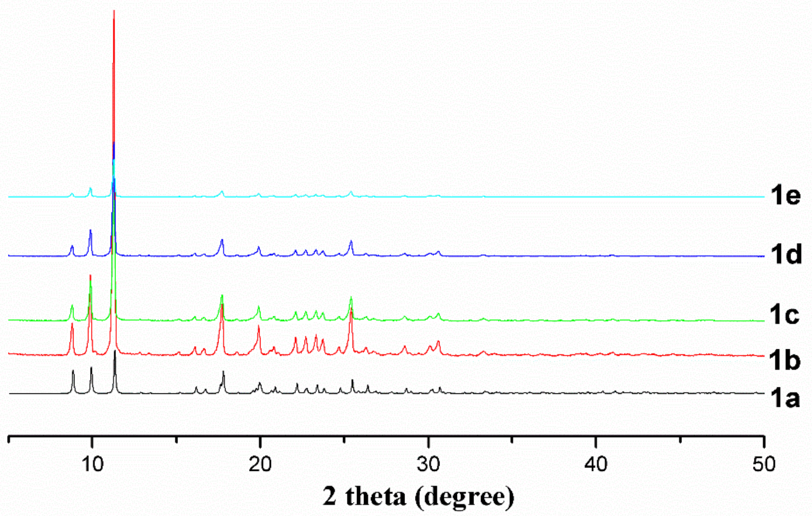
1e
1d
1c
1b
1a
10
20
30
40
50
2 theta (degree)

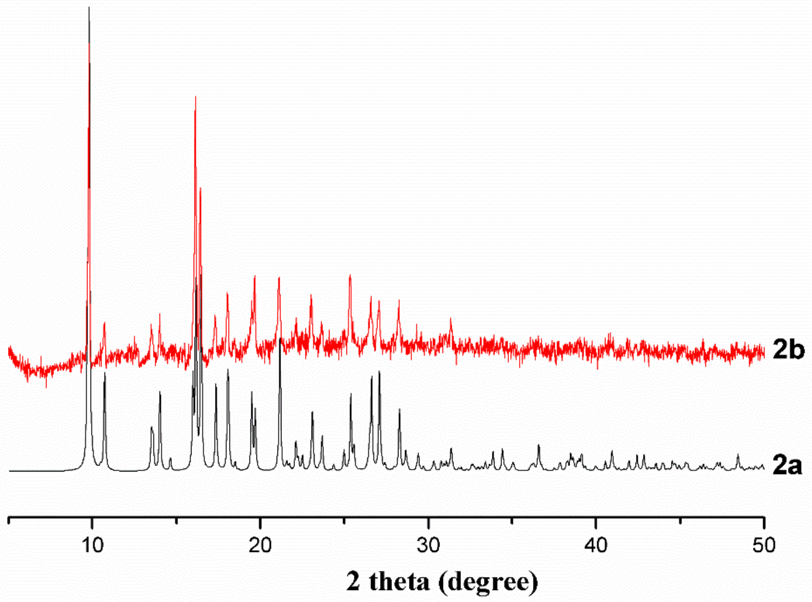
2b
2a
10
20
30
40
50
2 theta (degree)

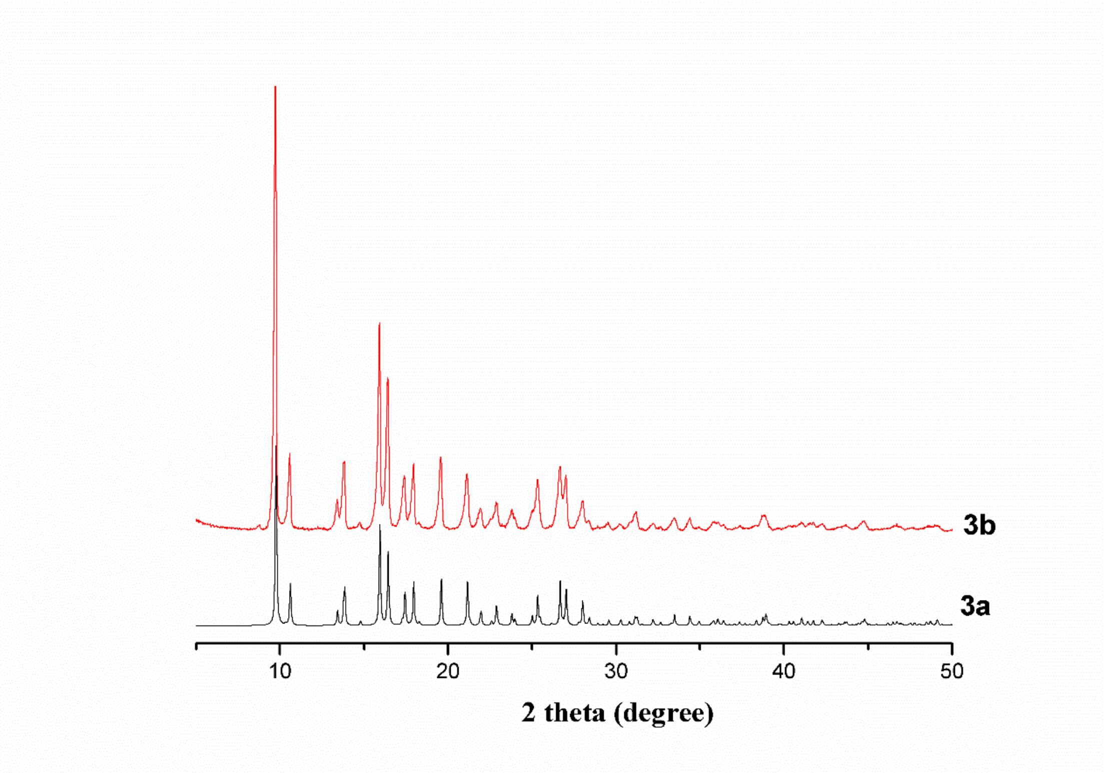

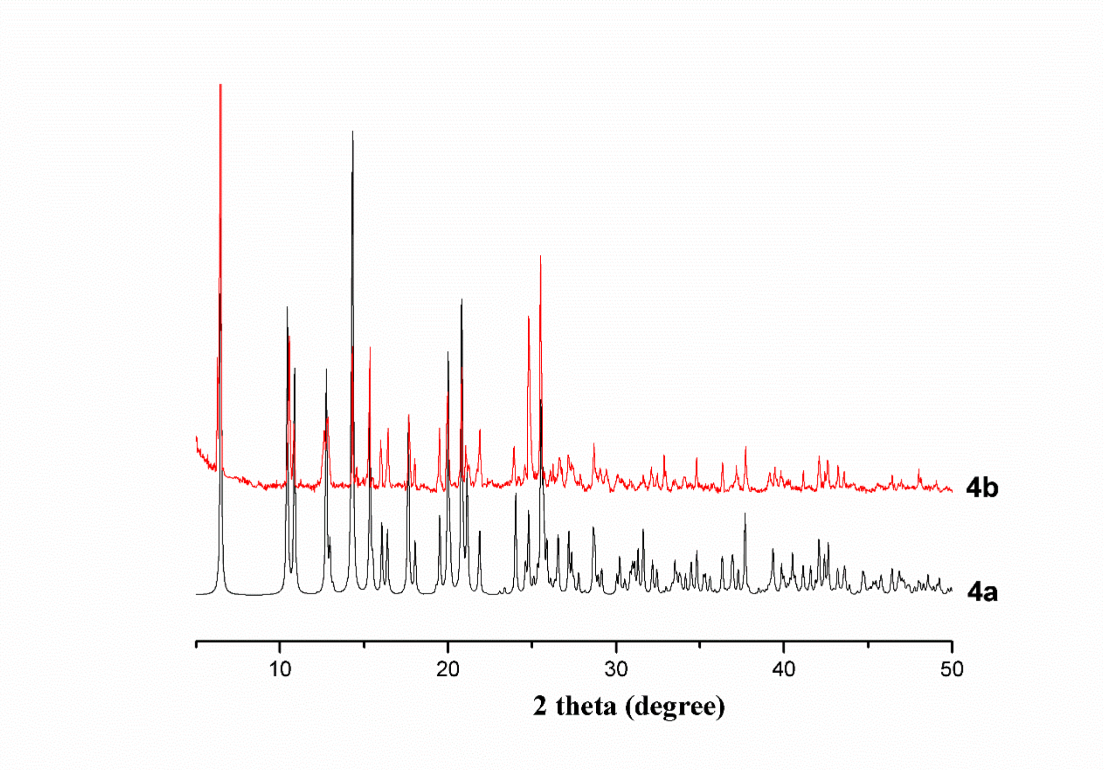

Figure S2. The X-ray powder diffraction patterns of complexes **1** – **4**: a – simulated; b – as-synthesized; c –the dehydrated phase at 160ºC; d –the dehydrated phase at 180ºC; e –the dehydrated phase at 200ºC.
